# Expression of novel acidic lipase from *Micrococcus luteus* in *Pichia pastoris* and its application in transesterification

**DOI:** 10.1186/s43141-021-00155-w

**Published:** 2021-04-07

**Authors:** Selfela Restu Adina, Antonius Suwanto, Anja Meryandini, Esti Puspitasari

**Affiliations:** 1 Graduate School of Microbiology, Department of Biology, Faculty of Mathematics and Natural Science, IPB University, Bogor, 16680 Indonesia; 2 Department of Biology, Faculty of Mathematics and Natural Science, IPB University, Bogor, 16680 Indonesia; 3 Department of Biotechnology Research and Development, PT Wilmar Benih Indonesia, Bekasi, 17530 Indonesia

**Keywords:** Lipase, Codon optimization, *Micrococcus luteus*, *Pichia pastoris*, Biodiesel

## Abstract

**Background:**

Lipases are promising biocatalysts for industrial applications and attract attention to be explored. A novel acidic lipase has been isolated from the lipolytic bacteria *Micrococcus luteus* EMP48-D (LipEMP48-D) screened from tempeh. The lipase gene had previously been overexpressed in *Escherichia coli* BL21, but the expression level obtained was relatively low. Here, to improve the expression level, the lipase gene was cloned to *Pichia pastoris.* We eliminated the native signal sequence of *M. luteus* and replaced it with α-mating factor (α-MF) signal sequence. We also optimized and synthesized the lipase gene based on codon preference in *P. pastoris*.

**Results:**

LipEMP48-D lipase was expressed as an extracellular protein. Codon optimization has been conducted for 20 codons, with the codon adaption index reaching 0.995. The highest extracellular lipase activity obtained reached 145.4 ± 4.8 U/mg under AOX1 promoter in *P. pastoris* KM71 strain, which was 9.7-fold higher than the previous activity in *E. coli*. LipEMP48-D showed the highest specific activity at pH 5.0 and stable within the pH range 3.0–5.0 at 40 °C. LipEMP48-D also has the capability of hydrolyzing various long-chain triglycerides, particularly olive oil (100%) followed by sunflower oil (88.5%). LipEMP48-D exhibited high tolerance for various polar organic solvents with low log *P*, such as isopropanol (115.7%) and butanol (114.6%). The metal ions (Na^+^, K^+^, Ca^2+^, Mg^2+^, Mn^+^) decreased enzyme activity up to 43.1%, while Fe^2+^ increased relative activity of enzymes up to 200%. The conversion of free fatty acid (FFA) into fatty acid methyl ester (FAME) was low around 2.95%.

**Conclusions:**

This study was the first to report overexpression of *Micrococcus* lipase in yeast. The extracellular expression of this acidic lipase could be potential for biocatalyst in industrial fields, especially organic synthesis, food industry, and production of biodiesel.

## Background

Lipases (triacylglycerol acyl-hydrolase, EC 3.1.1.3) are enzymes that have the responsibility to catalyze the hydrolysis of triglyceride ester bonds into diglycerides, monoglycerides, fatty acid, and glycerol, and also work for the synthesis of long fatty acid chains acylglycerol at the interface between oil and water [1–3]. Owing to their enzymatic properties, lipases have been widely utilized as biocatalysts and are an integral part of major industries, such as fats and oils, detergent formulation, biosensors, pharmaceutical, and biodiesel production [4–6]. The lipase used in those applications is selected based on its specificity and stability under an exceptional environment, for instance, high or low temperature, the presence of organic solvents, and alkaline or acidic conditions [7–9]. Lipases with resistance to acidic environments or acidic lipase become an important requirement for industrial practice, such as synthesizing isoamyl acetate in flavor industry [10], acid bating of fur and wool in textile industry [11], gastric digestion and feed purpose [12, 13], enzyme therapy, and oleochemical and biotechnological industry [14]. Accordingly, acidic lipase seems to command a huge market potential.

Notwithstanding the many lipase-producing strains that have been reported, the investigation on acidic lipase-producing strain is still a minority. Several acidic lipases have been studied to have optimum activity or stability under acidic conditions and have been isolated from *Aspergillus niger* NCIM 1207 [15], *Penicillium simplicissimum* [16], *A. niger* AN0512 [17], *A. niger* ANL [18, 19], *Pseudomonas gessardii* [14], *P. fluorescens* [20], *A. terreus* [21], *Enterococcus durans* NCIM5427 [22], *Candida viswanathii* [23], *Bacillus pumilus* [24], *B. subtilis* [25], *Neosartorya fischeri* P1 [26], *Meyerozyma guilliermondii* [13], and *Rasamsonia emersonii* [27].

Recently, the research about acidic lipase that has been reported is LipEMP48-D lipase, isolated from the lipolytic bacteria *Microccoccus luteus* EMP48-D screened from tempeh [28]. This lipase is relatively new because there has been no literature available for *M. luteus* lipase. *M. luteus* EMP48-D is the only actinobacteria that has been reported to produce acidic lipase and has been characterized so far. In the previous study, lipase gene from *M. luteus* EMP48-D has been cloned and overexpressed in *E. coli* BL21(DE3). However, the expression level obtained was relatively low due to the presence of native signal sequence *M. luteus* not recognized by *E. coli* system, so that overexpression might be manifested in the inclusion bodies. The overexpression product that accumulated as inclusion bodies and miss-folded in *E. coli* was obtained as soluble and correctly folded protein when expressed in the methylotrophic yeast, *P. pastoris* [29]. Besides, *P. pastoris* can be grown to very high cell densities using minimal media, has high levels of protein expression at intra- or extracellular, secretes low level of native proteins so it is easy for purification, has the ability to perform protein modification (appropriate folding and glycosylation), and has strong promoters [30–32]. It makes the *P. pastoris* become the most frequently used host for heterologous production of recombinant proteins [33].

Hence, to improve the expression level of the LipEMP48-D gene, the native signal sequence of *M. luteus* was eliminated from ORF of LipEMP48-D gene and replaced with α-MF signal sequence that has been within the *P. pastoris* vector. The lipase gene was adapted for *P. pastoris* expression machine according to the preferred codons of *P. pastoris*. The recombinant plasmid was constructed in pPIC9K and pPICZαA vectors and transformed into *P. pastoris* KM71 (Mut^S^) strain. Then, LipEMP48-D was characterized and assayed for transesterification activity.

## Methods

### Strains, vectors, media, and chemicals

*E. coli* DH5α (dlacZ Delta M15 Delta(lacZYA-argF) U169 recA1 endA1 hsdR17(rK-mK+) supE44 thi-1 gyrA96 relA1) was continually preserved in our laboratory. *P. pastoris* KM71 (his4 arg4 aox1∆::ARG4) was purchased from Invitrogen (USA). pPICZαA and pPIC9K vectors were purchased from Invitrogen (USA). Restriction enzymes (*Eco*RI, *Kpn*I, *Pme*I, *Sac*I, and *Xba*I) and T4 DNA ligase were from NEB (USA).

LB (1% peptone, 0.5% yeast extract, 1% NaCl, 100 μg/mL ampicillin) and LSLB (1% peptone, 0.5% yeast extract, 0.5% NaCl, and 25 μl/mL zeocin) were used for *E. coli* growth. YPDS Agar (1% yeast extract, 2% peptone, 2% dextrose, 1 M sorbitol, 1.5% agar) was used for the *P. pastoris* screening. YPD Broth (1% yeast extract, 2% peptone, 2% dextrose, and 2% agar) was used for the *P. pastoris* growth. BMGY (1% yeast extract, 2% peptone, 0.34% YNB, 4 × 10^−5^% biotin, 1% glycerol, and 100 mM potassium phosphate buffer pH 6.0) was used for the *P. pastoris* pre-induction growth medium. BMMY (1% yeast extract, 2% peptone, 0.34% YNB, 4 × 10^−5^% biotin, and 100 mM potassium phosphate buffer pH 6.0) was used for the *P. pastoris* induction medium.

### Isolation of the LipEMP48-D gene and signal sequence elimination

The source of gene encoding lipase from LipEMP48-D had previously been cloned into pGEM-T Easy vector and maintained in *E. coli* DH5α [28]. LipEMP48-D had been isolated originally from *M. luteus* [34]. LipEMP48-D gene was released from pGEM-T Easy vector using *Eco*RI and verified by using specific primers Lip013f (5′-CCC CGA CGC TAG CCG AG-3′) and Lip013r (5′-CAT CTG CAT CCG AGA GAC CG-3′), with procedure reaction as a denaturation at 95 °C for 5 min, 30 cycles (95 °C for 45 s, 56 °C for 30 s, 72 °C for 90 s), finally extension at 72 °C for 5 min. The PCR products were purified by using QIAquick Gel Extraction Kit Protocol from Qiagen (USA) and used for the next procedure.

The native signal sequence of LipEMP48-D was eliminated from the ORF of lipase gene (GenBank: MK618664.1) by using primers Lipml_KpnI-F (5′-TTA ATG GTA CCG CCC AGG AGT CGG CCC-3′) and Lipml_XbaI-R (5′-TTA ATT CTA GAG AAC CAC CCG CAC GAG TCG-3′). Primers were designed to carry the *Kpn*I restriction site at the 5′-end and *Xba*I at the 3′-end. The PCR reaction was performed for pre-denaturation at 94 °C for 5 min, followed by 30 cycles of 95 °C for 45 s, 67 °C for 60 s, and 72 °C for 90 s, and extension at 72 °C for 5 min. The lipase gene was digested by *Kpn*I and *Xba*I, and used as template sources (EMP-N) and control.

### Codon optimization and synthesis of the LipEMP48-D gene

The LipEMP48-D gene without native signal sequence (EMP-N) was optimized according to the codon usage preference of *P. pastoris* (http://www.kazusa.or.jp/codon/) by using the OPTIMIZER web-based server (http://genomes.urv.es/OPTIMIZER/) [35]. The optimized DNA sequence was synthesized by Gene Synthesis (Macrogen, South Korea). The DNA-synthesized product (EMP-S) was ligated into pTOP Blunt V2 vector. The specific primers, Emp2_EcoRI-F (5′-AAG CTG AAT TCC AAG AAT CTG CTC CAG CTC CAG ATG C-3′) and Emp2_EcoRI-R (5′-TAT ATG AAT TCA AAC CAA CCA CAA GAA TCA GCA CAC C-3′) were also designed with *Eco*RI restriction site for cloning preparation. The recombinant plasmid, pTOP+EMP-S, was used as the template for PCR amplification. The PCR reaction procedure was set as pre-denaturation step at 94 °C for 5 min, 30 cycles of 95 °C for 45 s, 67 °C for 60 s, 72 °C for 90 s, and followed by an extension step at 72 °C for 5 min. The purified PCR product was digested with *Eco*RI and used as template for the next methods.

### Construction of recombinant strains

The EMP-N was inserted into the multiple cloning sites of pPICZαA vector between *Kpn*I and *Xba*I restriction sites. The recombinant plasmid was verified by enzyme digestion and sequence analysis. The EMP-S was ligated into pPICZαA and pPIC9K vectors to construct recombinant plasmids. The recombinant plasmids were verified by enzyme digestion and sequence analysis.

### Transformation and production of protein

The ligated constructs were transformed by heat shock into *E. coli* DH5α cells. Then, the recombinant plasmids pPICZαA+EMP-N and pPICZαA*+*EMP-S were linearized by *Pme*I, and pPIC9K+EMP-S was linearized with *Sac*I. All of the recombinant plasmids were transformed into *P. pastoris* KM71 competent cells by electroporation (2 kV 400 Ω). The transformants were spread on selective YPDS plates containing zeocin (100 μl/mL) for pPICZαA recombinants and geneticin (0.25 mg/mL) for pPIC9K recombinants. Recombinant strains were picked up and kept on new YPDS plates that have been supplemented with antibiotics.

The seed culture and enzyme production were carried out based on *Pichia* Expression Kit protocol from Invitrogen (USA). The positive recombinant strains were grown on the YPD plates for 2–3 days. Then, 5 mL of the YPD medium was inoculated with a single colony and incubated at 30 °C and 225 rpm in 50 mL shake flasks for 18 h or until the OD_600_ reached 2–6. Seed culture (10%) was inoculated onto 50 mL BMGY medium (pH 6.0) at 30 °C and 225 rpm for 18 h. The cells were centrifugated at 4000×*g* at room temperature for 10 min. The cell pellet was resuspended in BMMY medium (pH 6.0). The culture was maintained at 30 °C, 225 rpm for 48 h, and every 24 h, methanol (1% v/v) was added to the culture to induce protein expression. The culture was harvested by centrifugation at 5000×*g* at 4 °C for 15 min. The supernatant formed was analyzed as crude extracellular enzyme extract. The recombinant protein was detected by 12% (v/v) SDS-PAGE.

### Assay of enzyme activity

The lipase activity was measured by alkali titration using olive oil as the substrate. Olive oil was emulsified with 4% w/v PVA at a ratio of 1:3 (v/v) for 2 × 3 min. The reaction mixture was composed of 5 ml of substrate, 0.5 ml of 150 mM citrate-phosphate (pH 5.0), and 0.75 ml of ddH_2_O, pre-incubated at 40 °C and 175 rpm for 5 min. Then, 0.25 ml of enzyme was added to the mixture and incubated at 175 rpm for 15 min under the optimum temperature of LipEMP48-D (40 °C) [28]. These conditions (40 °C, pH 5.0, 15 min) will be referred to as standard conditions. The reaction was stopped by the addition of 5 ml 95% ethanol solution. The reaction solution was titrated with 0.025 M NaOH standard solution. One unit of enzyme activity is defined as the amount of enzyme that released 1 μmol of fatty acids per minute. Specific enzyme activity was expressed as units per mg of protein.

### Characterization of the LipEMP48-D

The optimum pH of LipEMP48-D was investigated with pH ranging from pH 3.0–4.0 (citrate buffer 0.1 M), pH 5.0 (citrate-phosphate buffer 0.15 M), pH 6.0–7.0 (phosphate buffer 0.1 M), pH 8.0–9.0 (Tris-HCl buffer 0.1 M), and pH 10.0 (glycine-NaOH buffer 0.08 M) at 40 °C. The pH stability was evaluated by incubating the LipEMP48-D lipase in 3 various buffers (pH 3.0, pH 5.0, and pH 8.0) for 120 min at 40 °C. The samples were collected at different time intervals (30 min, 60 min, 90 min, and 120 min). The effect of organic solvents on LipEMP48-D stability was checked after pre-incubation of enzyme with organic solvents at a ratio 1:1 (v/v) for 15 min at 40 °C. Types of organic solvents used include methanol, ethanol, butanol, isopropanol, acetonitrile, n-hexane, and n-heptane. The effect of metal ions on LipEMP48-D lipase activity was determined by incubating the enzyme in 0.01 M solutions of NaCl, KCl, CaCl_2_, MgCl_2_, MnCl_2_, FeCl_2_, and FeSO_4_ for 15 min. The hydrolysis activity on various substrates was evaluated towards olive oil, corn oil, sunflower oil, canola oil, rice bran oil, palm oil, and soya oil. All triglycerides were adjusted to the emulsions. The activity was measured under the assay conditions as described above.

Each experiment was independently repeated three times. Student’s *t* test was used for the analysis of statistical significance (*P* value) in this study, and Minitab v.19.1.1 (USA) was used for these analyses. A *P* value of less than 0.05 was considered significant. Control was defined as enzyme activity without the treatment of each variable. Blanks were made according to the measurement conditions of each variable without the addition of the enzymes. The measurement results are displayed in the form of relative activity values for data on the effect of variables on enzyme activity and residual activity value for enzyme stability data.

### Transesterification activity of the LipEMP48-D

The transesterification activity of crude enzyme extract was measured based on the principle of the enzymatic synthesis of biodiesel from free fatty acids (FFAs). The types of FFAs used are palm fatty acid distillate (PFAD) and fatty matter (FM) which are regularly used in our laboratory. The fatty acid composition of PFAD was 45.9% of palmitic acid, 36.5% of oleic acid, and 10.3% of linoleic acid. The fatty acid composition of FM was 44.7% of palmitic acid, 37.9% of oleic acid, and 10.2% of linoleic acid.

Before use, the FFA was heated until it melts. Transesterification reaction for LipEMP48-D lipase was carried out in a 50-mL shake flask using a rotary orbital shaker. The reaction mixture is composed of 8 ml of FFA and 110 U/mg enzyme. Some stainless-steel beads were added to aid the stirring process during the reaction. The reaction mixture was incubated at 50 °C [36]; 1 mL of methanol was added every 1 h until the third hour and left for 5 h. FFA which has been successfully converted into fatty acid methyl ester (FAME) (biodiesel) was indicated by the formation of two layers in the shake flask.

## Results

### Optimization of LipEMP48-D lipase gene

**Fig. 1:**
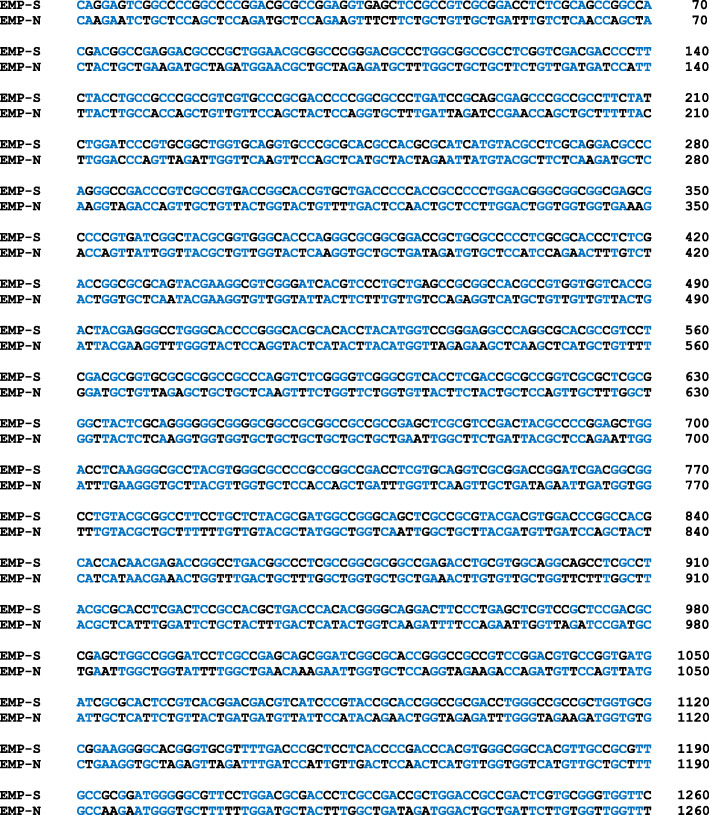
Alignment of sequence of LipEMP48-D native (EMP-N) and LipEMP48-D synthesis—codon optimization product (EMP-S). The blue highlight is identic bases

The entire ORF of the LipEMP48-D lipase gene consisted of 1356 bp encoding 451 amino acid residues, including 31 amino acids of native signal sequence of *M. luteus*. The native signal sequence was eliminated by *Eco*RI at the sites between 93 and 94 bases, it resulting in 1263 bp full ORF without signal sequence (Fig. 1). The LipEMP48-D gene without native signal sequence (EMP-N) was redesignated and synthesized as one amino acid-one codon based on the codon usage bias of *P. pastoris* without changing the encoded amino acid. Codon optimization has succeeded in optimizing the codon usage, which is shown as the effective number of codons (ENc) from 30 codons to 20 codons. This result automatically affects the codon adaptation index (CAI), which was indicated by an increase from 0.408 to 0.995. Sequence analysis showed that 432 bp from 1260 bp were effectively replaced. The new sequence of LipEMP48-D is 65.6% homologous to sequence before optimization, and the GC content was reduced from 76.5 to 44.7%. EMP-S was cloned into the pPIC9K and pPICZαA vectors and fused with α-MF signal sequence.

### Overexpression of LipEMP48-D in *P. pastoris*

**Fig. 2:**
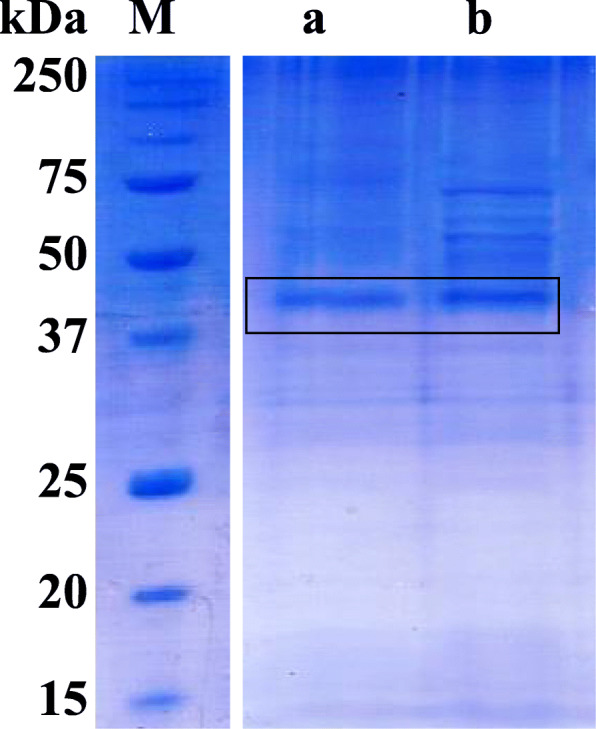
SDS-PAGE of the LipEMP48-D non-optimization (**a**) and codon optimization (**b**). Marker precision plus protein standards (biorad)

*P. pastoris* KM71 which have been integrated with the recombinant plasmid was cultivated in shake flask with 1% (v/v) methanol induction. The LipEMP48-D lipase was expressed as an extracellular protein. The highest lipase activity produced was 145.4 ± 4.8 U/mg protein in the supernatants after 48 h of methanol induction, considering the activity measured at standard condition. This expression was 9.7-fold higher than the previous activity in *E. coli*. The EMP-N only obtained 31.0 ± 1.1 U/mg, 4.8-fold lower than EMP-S also by using the *P. pastoris* KM71. The protein weight of both supernatants was detected by SDS-PAGE (Fig. 2). SDS-PAGE analysis displayed that the molecular weight was approximately 40 kDa; this molecular weight was similar for both of them.

### Characterization of the LipEMP48-D lipase

#### Effects of pH on LipEMP48-D activity and stability

**Fig. 3:**
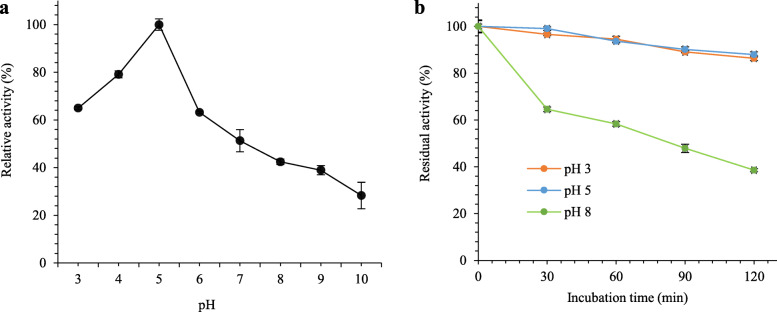
Optimal pH (**a**) and pH stability (**b**) of LipEMP48-D

The LipEMP48-D was characterized using alkali titration and olive oil emulsion as a substrate. The effect of pH on LipEM4P8-D activity and stability are displayed in Fig. 3. The LipEMP48-D represented maximal specific activity (100%) at pH 5.0 (Fig. 3a). The activity significantly decreased up to pH 6.0 and reducing continuously from pH 6.0 to pH 10.0. Among three conditions of pH has been investigated (pH 3.0, pH 5.0, and pH 8.0). The hydrolytic activity of LipEMP48-D had good stability at pH 5.0 and pH 3.0, as shown in Fig. 3b, resulted in the retention of more than 85% of the initial activity of LipEMP48-D after incubation for 120 min at 40 °C.

#### Effects of organic solvents on LipEMP48-D activity

**Fig. 4:**
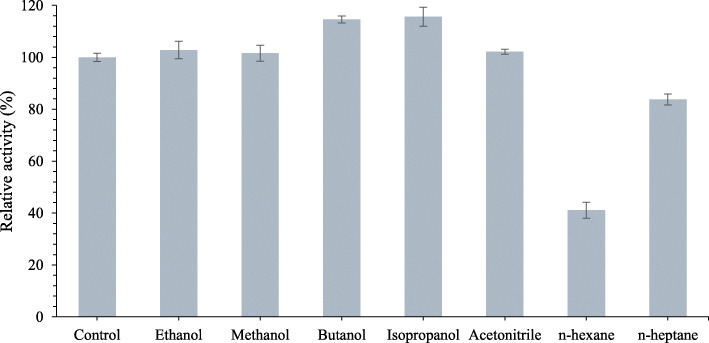
Effect of organic solvents on LipEMP48-D activity

The effect of organic solvent on LipEMP48-D activity is presented in Fig. 4. The LipEMP48-D exhibited high stability in the presence of hydrophilic organic solvents (low log P), such as isopropanol (115.7%), butanol (114.6%), ethanol (102.8%), acetonitrile (102.2%), and methanol (101.6%). Nevertheless, the hydrophobic solvents (high log *P*), like n-hexane and n-heptane, decreased their activity to 58.9% and 83.8%, respectively.

### Effects of various metal ions on LipEMP48-D activity

**Fig. 5:**
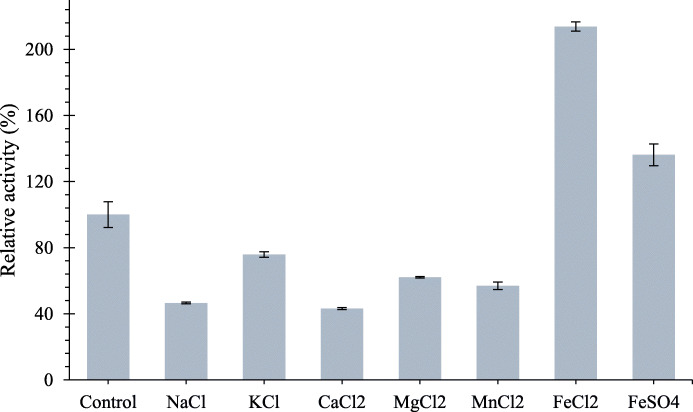
Effect of metal ions on LipEMP48-D activity

The relative activities of LipEMP48-D in several metal ions are shown in Fig. 5. The hydrolytic activity was increased up to 113.8% and 36.2% of its initial activity in the presence of 0.01 M concentration of FeCl_2_ and FeSO_4_, respectively. Under the same concentration, the lipase activity was slightly inhibited by the presence of K^+^ (24.1%), Mg^2+^ (37.9%), and Mn^+^ (43.1%), and strongly by Na^+^ (53.4%) and Ca^2+^ (56.9%).

### Hydrolysis activity on various natural oil substrates

**Fig. 6:**
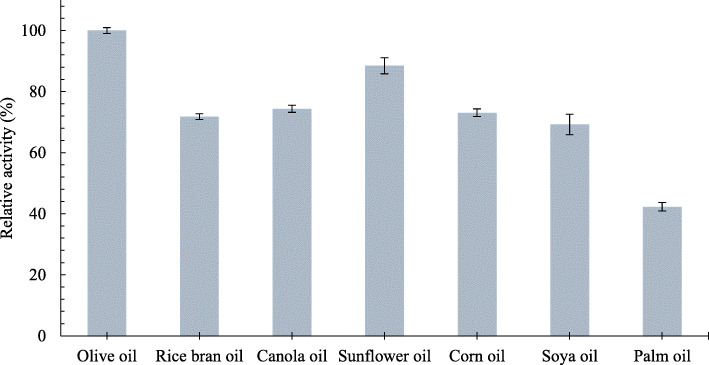
Comparison of hydrolysis capability by LipEMP48-D on various long-chain triglycerides

Some of long-chain triglycerides, as presented in Fig. 6, were capable to be hydrolyzed by LipEMP48-D. The enzyme exhibited the highest activity toward the olive oil (100%) and followed by sunflower oil (88.5%), canola oil (74.4%), corn oil (73.1%), rice bran oil (71.8%), soya oil (69.2%), and palm oil (42.3%).

### The potential of LipEMP48-D for biodiesel production

The potential of LipEMP48-D to produce biodiesel was measured according to the LipEMP48-D transesterification activity in converting FFA from the substrate to FAME. The LipEMP48-D only managed to convert the FFA of PFAD was 1.06%, which was from 47.68% into 44.73% after 5 h of transesterification reaction. Meanwhile, FFA conversion was 2.95%, from 85.06% to 83.99% was obtained after 5 h transesterification reaction between FM and methanol.

## Discussion

Acidic lipases have the potential for different industrial applications. However, there has not been much investigation about acidic lipases. LipEMP48-D is a novel acidic lipase derived from the lipolytic bacteria *M. luteus*. In order to improve the expression level, the lipase gene was cloned in *P. pastoris. P. pastoris* has become a substantial workhorse for biotechnology, especially for heterologous lipase protein production [7, 37, 38]. The first strategy was to eliminate the native signal sequence of *M. luteus* and replace it with α-mating factor (α-MF) signal sequence that was commonly within the *P. pastoris* expression vectors. The use of α-MF peptide signals succeeded in translocating LipEMP48-D lipase protein out of *P. pastoris* cells.

Most amino acids are encoded by more than one codon; thus, the codon optimization aim to avoid the use of rare codons [39]. Protein expression and codon bias are linked, also correspond to abundant tRNAs [40, 41]. Each organism has its codon usage. Between *P. pastoris* and *M. luteus* had gaps in codon usage, which may cause a mismatch codon *M. luteus* in *P. pastoris*. Codon optimized and synthesized the lipase gene base on *P. pastoris* codon preference which was a strategy to optimize the LipEMP48-D; thus, it became more recognizable to *P. pastoris*. Codon optimization is a gene design engineering to improve recombinant genes without altering the function and sequence of amino acids [41]. There are two main standards for codon optimization, i.e., ENc and CAI. The ENc value ranges from 20 to 61, the smaller the ENc value, the more effective the use of codons, vice versa [42, 43]. The CAI is the average probability of occurrence of a codon encoding an amino acid [44]. The greater the CAI value of the LipEMP48-D sequence, the greater the chances of the codon being translated correctly in the heterologous host *P. pastoris*. As previously described in the result, the codon optimization has been effectively optimized close to its maximum value.

*P. pastoris* that has been integrated with recombinant plasmid was assayed for hydrolytic activity. The highest specific activity was produced by *P. pastoris* KM71 strain which was integrated with the pPIC9K+EMP-S which had been optimized for codon preference. These results indicate an increase in the LipEMP48-D expression about a 9.68-fold compared to the previous expression in *E. coli* BL21. The result also showed that the expression of LipEMP48-D without codon optimization was relatively low, but also managed to increase the activity about a 2-fold (52%) than before. This condition is assumed because the codon optimization sequence is more recognized by the *P. pastoris* system compared with non-optimized, so that the translation process runs correctly. The use of rare codons usually does not affect translation rate but observed protein misfolding and aggregation [45]. The enhanced expression of LipEMP48-D might in consequence of protein lipase be folded correctly so it can be secreted as a functional mature protein. Optimization of codon has also been reported to be successful in *Yarrowia lipolytica* LIP2 lipases [46] and *Galactomyces geotrichum* mafic-0601 lipases with an increase in activity of 48.7% [47].

LipEMP48-D had a good performance under acidic conditions (Fig. 3) So far, there has not yet been any information regarding the optimum pH performance of *M. luteus* lipase. Lipases from the same genus, *M. flavus* [48] and *M. roseus* [49], both exhibited optimum conditions at high pH (pH 8.0). This fact shows that LipEMP48-D lipase has its character compared to lipase in the same genus. As shown in Fig. 3b, LipEMP48-D had high stability under acidic conditions. This property is desirable for many industrial applications, especially for biodiesel production from crude or residual vegetable oil, which has presence of high FFA contents. Furthermore LipEMP48-D lipase activity tends to be better in hydrolyzing olive oil and other vegetable oils which are dominated by unsaturated fatty acids, oleic acid (C18:1) and linoleic acid (C18:2), than palm oil which contains a nearly balance content of palmitic between palmitic acid (C16:1) and oleic (C18:1). Basically, LipEMP48-D lipase has an almost as good capability in the hydrolysis of C16 and C18 substrates [28]. But in this case, it was assumed that the unsaturated fatty acids resulting from the hydrolysis reaction were more detectable during the acid-base titration than the saturated fatty acids. Unsaturated fatty acids have better polarity than saturated fatty acids. Even though their solubility is equally good in ethanol, the relatively high melting point of palmitic acid compared to oleic and linoleic acids makes it less detectable or possibly undetectable at the time of titration. The lipase activity of 42.3% was detected from the reaction using palm oil as a substrate, presumably consisting of unsaturated fatty acids produced in the hydrolysis reaction and was detected properly at the time of titration. The fatty acid content of palm oil consists of 50% saturated fatty acids, consisting of 44% palmitic acid (C16:0) and 5% stearic acid (C18:0), while unsaturated fatty acids consist of 40% oleic acid (C18:1) and 10% linoleic acid (C18:2), and linolenic acid (C18:3) [50, 51].

LipEMP48-D has exhibited high stability in the presence of hydrophilic organic solvents. LipEMP48-D even showed increased activity in many polar organic solvents. The same characteristics were reported in another acidic lipase from *A. niger* AN0512, which has a high tolerance for various polar organic solvents with log *P* < 0.8 [8] and *C. viswanathii* lipase, has a well-performed in methanol [23]. The stability of lipase in organic solvents is an essential prerequisite for their applications in organic synthesis [52]. Different lipases have different sensitivity to solvents [53]. Although the stability of non-polar solvents has been reported for most lipases [54], there are very few reports on the activation of lipases in polar solvents [17]. In this study, lipase was activated by various polar organic solvents, such as isopropanol, butanol, methanol, ethanol, and acetonitrile.

The metal ions Fe^2+^ stimulated the hydrolysis activity of LipEMP48-D, but it is not a cofactor of LipEMP48-D. This is evidenced by the maintained relative activity of LipEMP48-D (68.6%) even though the metal ions that bind to the enzyme have been chelated using 10 mM EDTA [28]. The increased activity due to the addition of FeSO_4_ and FeCl_2_ may be due to the formation of a complex between the Fe^2+^ ion and the ionized free fatty acids. The complex formed can reduce the solubility of free fatty acids (products) in the lipid-water interface resulting in them being freed more easily from the catalytic site of the enzyme so that there are openings that can carry out the next reaction [55]. On addition of metal ions, the fatty acids form the corresponding metal salts. In the case of divalent ions, it is likely that the cations can form di-salts and salts. They inhibit the activity of lipase and also hinder the hydrolysis reaction [56].

The conversions of FFAs have been shown in the result, indicated that the ability of LipEMP48-D to produce biodiesel in the form of FAME was still low. The slight accumulation of biodiesel that was formed is thought to be due to the relatively low activity of LipEMP48-D, about 110 U/mg of which was used for biodiesel conversion. It is because the FFA from PFAD and FM cannot be converted completely, especially in a relatively short time. The substrate used in this case was PFAD and FM. PFAD is a byproduct of the production of crude palm oil, which has consists of more than 80% FFA with the main compound was palmitic acid and oleic acid [57]. FM is a byproduct of biodiesel production using the chemical catalyst CH_3_ONa which has a high moisture content. The FFA content has lower than PFAD, approximately 47.68%.

The biodiesel production by lipase enzymatic transesterification is affected by several factors, including the enzyme activity, concentration of substrate, the variety of fatty acids present in the substrate, water activity, pH, reaction temperature, and the distance between the enzyme molecule and the substrate [5, 58–60]. Beyond that, lipase is more favorable compared with chemical catalysts. Many studies have also reported on the good performance of lipase in producing biodiesel. Unfortunately, all these studies that have been reported are alkaline lipases. There have been no studies on the ability of acidic lipases in converting FFA to produce biodiesel, for a comparable comparison with LipEMP48-D. As previously explained, acidic lipase has potential to be developed further; it can be applied especially in industries that require acidic conditions. One of them is in the production of biodiesel from crude or residual vegetable oil, in which the presence of high FFA content automatically causes very acidic conditions at the beginning of the reaction. Of course, under these conditions, the use of acidic lipases may be better than alkaline lipases.

## Conclusions

Our study indicated that combined codon optimization and optimization of signal sequence for extracellular expression of LipEMP48-D are an effective method to improve the production of LipEMP48-D in *P. pastoris*. LipEMP48-D is one of few unique acidic lipases which exhibited high tolerance to high concentrations of polar organic solvents and also capable to hydrolyze various long-chain triglycerides. Furthermore, our results presented here will greatly contribute to improve the production of recombinant proteins in *P. pastoris* and offer a greater value for biocatalyst, especially in organic synthesis, food industry, and biodiesel production.

## Data Availability

The authors declare that all generated and analyzed data have been included in the article.

## Abbreviations

ORF: Open reading frame
LB: Luria Bertani Broth
LSLB: Low-salt Luria Bertani Broth
SDS-PAGE: Sodium dodecyl sulphate-polyacrylamide gel electrophoresis
NaOH: Sodium hydroxide
PVA: Polyvinyl alcohol
FFA: Free fatty acid
FAME: Fatty acid methyl ester
PFAD: Palm fatty acid distillate
NaCl: Sodium chloride
KCl: Potassium chloride
FeCl_2_: Ferrous chloride
FeSO_4_: Ferrous sulfate
CaCl_2_: Calcium chloride
MgCl_2_: Magnesium dichloride
MnCl_2_: Manganese (II) chloride
CH_3_ONa: Sodium methoxide
